# An Evaluation of the Clotting Properties of Three Plant Rennets in the Milks of Different Animal Species

**DOI:** 10.3390/foods8120600

**Published:** 2019-11-20

**Authors:** Katia Liburdi, Carlo Boselli, Gilberto Giangolini, Simonetta Amatiste, Marco Esti

**Affiliations:** 1 Department of Agricultural and Forestry Sciences (DAFNE), Tuscia University, Via San Camillo de Lellis, 01100 Viterbo, Italy; esti@unitus.it; 2 Experimental Zooprophylactic Institute Lazio and Toscana “Mariano Aleandri”, Via Appia Nuova 1411, 00178 Rome, Italy; carlo.boselli@izslt.it (C.B.); gilberto.giangolini@izslt.it (G.G.); simonetta.amatiste@izslt.it (S.A.)

**Keywords:** calf rennet, clotting parameters, curd firming, milk coagulation, vegetable coagulants

## Abstract

*Cynara cardunculus*, *Carica papaya* and *Ficus carica* extracts are proposed as milk coagulants herein. Their coagulation efficiency was measured in bovine, buffalo, goat and sheep milk incubated at different temperatures. The milk-clotting and proteolytic activities as well as the lactodynamographic parameters were determined considering animal rennet as a reference coagulant. The vegetable coagulant, extracted from *C. cardunculus* pistils, proved to be the most suitable milk-clotting enzyme for cheesemaking, since it possesses similar milk clotting properties to conventional calf rennet. *F. carica* latex, but seemed to be a promising alternative coagulant at higher temperatures. The strong proteolytic activity of papain caused poor milk coagulation in all milk samples. To conclude, this result also supports the original hypothesis of this study that the excessive proteolytic nature of plant coagulants can negatively affect the cheesemaking process. The optimization of using a plant rennet in a dairy application can be done by selecting the appropriate plant rennet with a consistent clotting efficiency. These innovative manufacturing processes may also lead to the optimization and production of new cheese varieties.

## 1. Introduction

The first essential biochemical process during cheesemaking is to convert liquid milk into a soft gel called curd. Ancient cheesemakers discovered that curd formation is induced by a substance present in the abomasum of ruminants. Therefore, since the onset of industrial cheese production, specialized agricultural enterprises have supplied cheesemakers with dried calf or lamb stomachs, or, later in the 20th century, concentrated or dried extracts of them (i.e., liquid or powdered rennet) in order to reduce the risks related to production and to ensure more hygienic manufacturing conditions [1]. Cheese production increased by a factor of 3.5 since 1961 but the rennet supply decreased due to the limited availability of ruminant stomachs [2]. Various factors such as the high price of rennet, religious concerns (e.g., Islam and Judaism), diet (vegetarianism) or a ban on the use of recombinant calf rennet uses (in France, Germany and The Netherlands) have encouraged the search for alternative milk-clotting sources [3]. Huppertz et al., [4] reported that alternative coagulants should have similar biochemical properties to those of calf rennet, such as high milk clotting power; high specificity toward κ-casein; proteolytic activity at cheesemaking pH and temperature; and a sufficient thermolability to ensure whey products without residual coagulant activity. The most commonly used rennet substitutes include enzymes of microbial origin and recombinant proteases metabolised by genetically modified microorganisms. These commercial milk-clotting enzymes are aspartic proteases (EC 3.4.23.) which cleave the Phe^105^–Met^106^ bond of bovine κ-casein. However, microbial coagulants may lead to off-flavour generation and affect cheese yield and quality [5,6]. Numerous studies have focused on using plant extracts as rennet substitutes [7,8,9,10]. Besides, these vegetable coagulants having the capacity to coagulate milk also have proteolytic activity. Proteases, such as ficin from *Ficus* sp. latex, papain from *Carica papaya* and cardosins from *Cynara* sp., are sometimes constituents of latex, fruits, roots, seeds and/or sap, but mainly of their leaves or flowers. More specifically, papain and ficin, classified as cysteine proteases, have a digestive proteolytic activity while aspartic proteases from cardoon have a specific activity for the Phe^105^–Met^106^ bond of bovine κ-casein [3]. Moreover, Silvestre et al. [11] reported as the optimum temperature for the clotting and proteolytic activities of vegetable extracts depends on several factors, such as the plant source, tissue, concentration and type of protease.

Vegetable extracts have been used as coagulants in cheesemaking since ancient times in the Mediterranean, West African and southern European countries [3]. Cheeses made with cardoon extract from *Cynara cardunculus* produced at artisanal level are granted a Protected Designation of Origin (PDO) in Spain and Portugal [12]. However, plant proteases have been isolated from several plant sources and studied for their milk-clotting ability [13]. The effects of plant coagulants extracted from *Ficus carica* latex, and *Carica papaya* on bovine milk have been studied by comparing their milk-clotting properties and enzymatic proteolysis products to those of commercial calf rennet [14]. Nevertheless, plant coagulants are seldom used in cheesemaking due to their strong proteolytic activity/milk-clotting activity [15] that causes lower yields of cheese, bitter flavours and texture defects (e.g., softness) [16]. However, cardoon extract has been used successfully for making ovine cheeses, but it produced poor quality cheese when used for coagulating bovine milk [14,17,18]. Caprifig latex is used in southern Italy for manufacturing Cacioricotta, a craft cheese made from overheated goat’s milk [19,20]. Several authors have reported on how the type of milk (bovine, ovine or caprine) and milk composition influence coagulum development and how they affect yield and cheese quality [17,21,22]. Significant effects were observed for the type of milk (bovine, ovine and caprine) when cardoon extract was used for milk coagulation [23,24].

The aim of this study was to evaluate the milk-clotting and proteolytic activities of three vegetable coagulants *(Cynara cardunculus, Carica papaya* and *Ficus carica*) in terms of type of milk (bovine, buffalo, goat and sheep) and temperature effects. The traditional animal rennet chymosin was used as reference coagulant. Another goal of the study was to measure traditional milk coagulation properties (lactodynamographic parameters (LAT): rennet coagulation time (r, min), curd firming time (k_20_, min) and curd firmness (a_60_, mm)) in order to better describe the variability of coagulation, curd firming and syneresis processes of the different milk samples and vegetable coagulants.

## 2. Materials and Methods

### 2.1. Materials

All of the reagents and commercial papain preparations from *Carica papaya* latex (EC 3.4.22.2) were purchased from Sigma-Aldrich Ltd. (Milano, Italy) unless otherwise stated.

#### 2.1.1. Calf Rennet

The calf rennet (Cr) used in this study was a commercial liquid preparation (Caglio Clerici, 175 International Milk Clotting Units (IMCU) mL^−1^) supplied by Caglificio Clerici, Como, Italy. 

#### 2.1.2. Preparation of Vegetable Coagulants

Cardoon extracts (Cc) were prepared using the protocol described by Liburdi et al. [12] (page 2), by soaking 6 g of stylets and stigmae of dried *C. cardunculus* flowers (harvested in July 2018) in 80 mL of distilled water for 20 min. Following vacuum centrifugation at 4 °C for 10 min at 7000× *g* (Beckman J2-21 centrifuge with rotor JA20.1; Beckman Coulter_®_, California, USA), the supernatant was collected and frozen at −20 °C using threalose as the protein stabilizer. It was then lyophilized in a VirTis AdVantage freeze-dryer (The VirTis Company, Gardiner, NY, USA) where all samples were cooled to a shelf temperature of −55 °C for 6 h, followed by primary drying at −15 °C for 40 h (80 mTorr chamber pressure). Fresh fig latex (Fc) was extracted from the unripe green fruits of *F. carica* L. tree cultivated in the Botanical Garden of Tuscia University (Viterbo) and collected in a clean tube containing 0.05% NaN_3_. All of the latex samples used in this study were collected in the early morning at the end of August 2018. The latex fluid was transported to the laboratory, diluted with distilled water (1:500), mixed and centrifuged at 19,000× *g* (Beckman J2-21 centrifuge with rotor JA20.1) and stored at −20 °C until used.

When required, a 5% (*w*/*v*) papain solution (Cp) was prepared dissolving the commercial preparation in distilled water. 

#### 2.1.3. Milk Samples

Individual milk samples from bovine, buffalo, goat and sheep herds were collected from farms located in the Lazio Region in October 2018. The milk samples were collected at morning milking and immediately transferred at 4 °C to the laboratory of the Istituto Zooprofilattico Sperimentale del Lazio e della Toscana “Mariano Aleandri” (Rome, Italy), where all milk samples were thermised (55 °C for 15 s). After that, the fat, protein, casein, lactose (MilkoScan FT6000; Foss, Hillerød, Denmark) and lactodynamographic analyses (Formagraph; Italian Foss Electric, Padova, Italy) were conducted. An aliquot of each milk sample was immediately transferred (at 4 °C) to the laboratory at Tuscia University for milk-clotting activity measurements. 

### 2.2. Experimental Procedures

#### 2.2.1. Determination of Protein Content

Total protein quantification of each plant coagulant (Cc, Cp and Fc) and the calf rennet was determined by the Bradford method [25]. In this assay, a series of BSA standard solutions (0.1–1.2 mg/mL) was prepared for calibration curve determination. The samples were incubated in the dark for 45 min and the absorbance was read at 595 nm (Shimadzu; Kyoto, Japan).

#### 2.2.2. Caseinolytic Activity 

Casein hydrolysis by proteases from the three vegetable extracts (Cc, Cp and Fc) were determined according to Anusha et al. [26]. In total 0.25 mL of each coagulant solution was incubated with 0.25 mL of substrate (1% *w*/*v* bovine casein in 0.05 M NaOH) and 0.5 mL of 0.1 M phosphate buffer (pH 6.5) for 20 min at different temperatures (25, 37, 50, 60 and 70 °C). The reaction was stopped by adding 0.5 mL of 15% (*w*/*v*) trichloroacetic acid and was then allowed to stand for 25 min at room temperature and centrifuged. Caseinolytic activity (CA) was assayed according to the colorimetric method developed by Anson [27] and Folin and Cicalteu [28]. Seven-point-five millilitres of NaOH (0.5 M) and Folin Ciocalteau reagent were diluted 1:2 with distilled water and added to 0.5 mL of supernatant and incubated for 20 min in the dark. The absorbance of the supernatant was measured at 660 nm with a UV-Vis spectrophotometer. One unit of the CA was defined as the amount of the enzyme that liberated 1 μg of tyrosine under standard assay conditions (acetate buffer at pH 6.5 for 20 min).

#### 2.2.3. Milk Clotting Activity 

The milk clotting activity (MCA) was evaluated for the Cc, Cp and Fc samples as reported by Anusha et al. [26], for bovine, buffalo, goat and sheep milks. MCA was assayed by adding 0.5 mL of the coagulant solution to 1.5 mL of milk incubated at different temperatures (25, 37, 50, 60 and 70 °C). The assay was performed in triplicate and coagulation times lower than 60 min were considered positive and included in the data set for the temperature effect on MCA. One unit of milk-clotting activity was defined as the quantity of protein required to coagulate 1 mL of milk in 40 min (2400 s) at the temperature evaluated:

(1) MCA, U/mgBSA=2400T×SEmgBSA

where *T* = time required for curd formation (seconds), *S* = volume of milk (mL) and *E* = volume of the coagulant (mL).

The milk clotting index, that is, the ratio of milk-clotting activity (MCA) to caseinolytic activity (CA), was calculated as given below:

(2) MCI=MCACA.

The CA and MCA relative activities ((%) relative CA and (%) relative MCA, respectively) were calculated considering the ratio of the activity at the optimum temperature to the activity at each temperature value.

#### 2.2.4. Lactodynamographic Analysis

The milk coagulation properties [29] were determined using a Formagraph (Italian Foss Electric, Padova, Italy) and 10 mL of each milk sample. The amounts of calf rennet and plant coagulant were first standardized against each milk, considering the results obtained in MCA experiment, so that the rennet clotting time for each coagulant was approximately within the range of 10–40 min. Moreover, the volume of each coagulant was made up to 1 mL and then added to the milk substrate and measured at 35 °C. Considering the longer curdling time required for the plant extracts, the time measurements were extended for up to 90 min and the following lactodynamographic parameters (LAT) were obtained:

*r* = clotting time: the time in minutes from the addition of rennet to the beginning of coagulation;
*k20* = curd firming: the time in minutes required until the curd was firm enough to cut; i.e., the width of the diagram equals 20 mm.
*a60* = curd firmness (mm) measured 60 min after adding the rennet.

### 2.3. Statistical Analysis

A two-way analysis of variance (ANOVA) was performed using statistical software (IBM-SPSS v19). The data obtained from the average of three replicate measurements were analysed for statistical significance using: (i) two-way ANOVA for testing the effects of the single factors (coagulant and type of milk) and their interactions with the milk clotting index (MCI) and LAT parameters (*p* < 0.01); (ii) one-way ANOVA to test for significant differences considering each independent factor (coagulant, milk samples and temperature). Tukey’s comparison was used to determine the significance between groups. A *p*-value ≤ 0.05 was considered significant. The results are reported as the means ± standard deviations.

## 3. Results and Discussions

The chemical compositions of bovine, buffalo, goat and sheep milks are reported in Table 1. The data shows that all samples were of good-to-average quality and variable in terms of casein, fat and protein contents. It is worth noting that the parameters measured are in accordance with data reported by other authors [30,31,32]. 

### 3.1. Milk-Clotting (MCA) and Caseinolytic Activities (CA) of Plant Extracts

**Figure 1:**
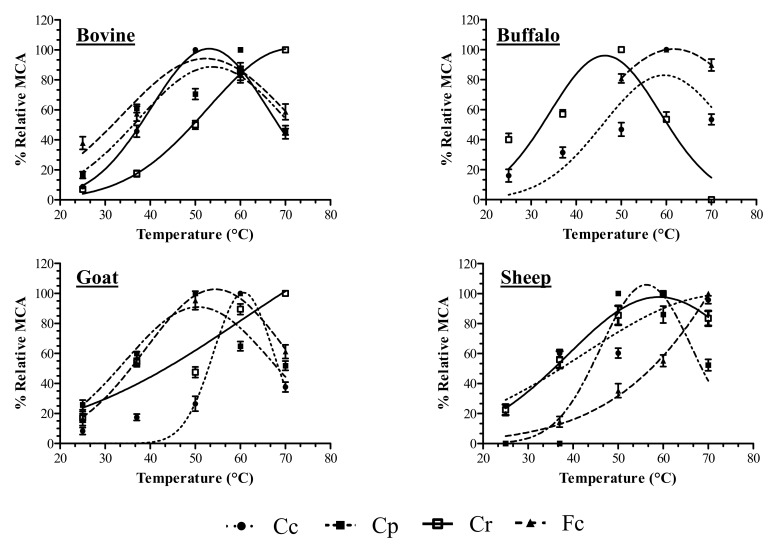
Temperature effect on the milk-clotting activity (MCA) of *Cynara cardunculus* (Cc), *Carica papaya* (Cp), calf rennet (Cr) and *Ficus carica* (Fc) extracts, measured in bovine, buffalo, goat and sheep milks. The percentage (%) relative MCA represents the mean of three independent determinations performed in triplicated for each coagulant.

In the first phase of the experiments, vegetable coagulants were tested for their MCAs in different milk types of milk at different temperatures (Figure 1). The maximum MCA value of the Cc sample was observed in bovine milk at approximately 50 °C, whereas the maximum MCA values of the buffalo, goat and sheep milks were observed at 60 °C. These findings are in line with previous studies that reported optimal coagulation activity of the *C. cardunculus* extract at temperatures ranging between 50 °C and 70 °C [33,34,35]. The Cp coagulant exhibited maximal MCA values in goat and bovine milk samples at 50 °C and 60 °C respectively. However, when Cp was added to sheep’s milk, coagulation only occurred at temperatures above 50 °C which corresponds to 100% MCA. Moreover, no visible coagulation effects were observed at any temperature when buffalo milk was coagulated by Cp. As reported by several authors [36,37], papain proved to have poor milk clotting abilities when used to coagulate bovine and caprine milk. *F. carica* latex exhibited good coagulant activity in all types of milk, reaching the maximum value at 50 °C in bovine milk, 60 °C in buffalo and goat’s milk and 70 °C in sheep’s milk. However, a low coagulation efficiency was observed when *F. carica* latex was used to coagulate buffalo and sheep milk at temperatures of 25–37 and 25 °C, respectively. These data are consistent with the findings of Nouani et al. [38] who demonstrated the thermophilic nature of *F. carica* extract. This is also confirmed by the fact that caprifig latex is used for producing the Italian “Cacioricotta” cheese which is made from overheated goat’s milk [16] (page 3). Finally, the MCA of Cr in bovine and goat samples increased with high temperatures, reaching its maximum value at 70 °C. A similar trend was observed in the sheep sample where 50 °C proved to be the optimum temperature for milk coagulation even if Cr maintained approximately 80% of its MCA at 70 °C. Only in buffalo milk, animal rennet exhibited a conventional bell-shaped curve with an optimum MCA value at 50 °C. The Cr results are consistent with those obtained by Rogelj et al. [39], who reported that milk coagulation performed with animal chymosin tends to be more efficient and heat-resistant at high temperatures. As expected, differences in MCA activity were observed between the three plant extracts and the calf rennet. The optimum milk coagulation temperature for vegetable extracts depends on several factors, such as the plant source, tissue, concentration and type of protease in the extracts. It is well known that *C. cardunculus* coagulant contains two enzymes, cardosin A and cardosin B, which are similar to chymosin and pepsin in terms of casein hydrolysis [40] while ficin isolated from the latex of various *Ficus* species possesses certain milk coagulation properties. Interestingly, ficin represents a heterogeneous protein fraction of fig latex, which includes at least five different isoforms with different milk clotting properties and caseinolytic activities [38,41] (page 8). 

**Figure 2:**
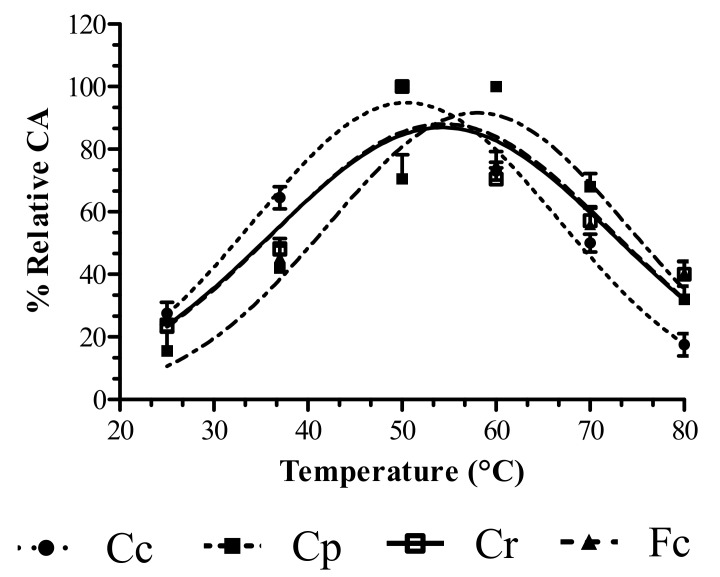
Temperature’s effect on the caseinolytic activities (CAs) of *C. cardunculus* (Cc), *C. papaya* (Cp), calf rennet (Cr) and *F. carica* (Fc) extracts, measured in buffer acetate (pH 5.5). The percentages (%) of relative CA represent the means of three independent determinations performed in triplicate for each coagulant.

In order to determine the MCA/CA ratio, which is essential for a plant extract’s application in cheesemaking, the caseinolytic activities of Cc, Cp and Fc was evaluated at various temperatures (Figure 2). As expected, the CA of animal rennet was lower than the CA of the plant extracts, while Cp showed higher levels of proteolytic activity with casein compared to Cc and Fc (data not shown). However, the CA profile proved to be affected by temperature for all of the coagulants analysed; the optimum values were 60 °C for Cp and 50 °C for Cc, Cr and Fc. Since rennin or rennin-like enzymes suitable for cheese production are characterized by high caseinolytic activity and low proteolytic activity, the MCA/CA ratio defined as MCI was considered in this research. A coagulant with a higher MCI is usually able to form curd, produce higher yields and fewer bitter peptides during cheese aging. However, low MCI values may result in lower raw cheese yields, poor curd firmness and the release of bitter peptides which affects the sensory attributes of the final product [42]. 

According to the two-way ANOVA (milk animal sources versus coagulants), MCI was significantly affected by the factors under study (*p* < 0.01, data not shown). Moreover, one-way ANOVA (Table 2) carried out on the data showed significant differences (Tukey’s HSD test *p* < 0.05, identified with the lower case letters) between the coagulants and the type of animal milk. Regardless of the type of rennet used, Cr showed a much higher MCA/CA ratio than those obtained for Cc, Cp and Fc coagulants for all types of animal milk at all temperatures. Considering the higher values, animal rennet obtained MCI values 23, 28 and 44 times higher than those obtained for *C. cardunculus*, *C. papaya* and *F. carica* extracts, respectively. As expected, differences in MCI were found between the three plant extracts evaluated at different temperatures for each milk sample. In bovine milk, the highest MCI values for Cp and Fc were obtained at 50 °C and 25 °C, respectively. In buffalo milk, the MCI values could not be calculated due to the absence of MCA for Cp at all temperatures and Fc at 25 and 37 °C. However, Cc proved to be the best of all of the vegetable extracts tested in buffalo milk. Cc and Cp obtained the highest values in goat’s milk at 60 and 37 °C, respectively, while the highest MCI values were only recorded for Cc in sheep’s milk at all temperatures, confirming that it can be efficiently used for manufacturing ewe’s milk cheese.

Plant coagulants have long been considered as possible substitutes for chymosin in the cheese-making process, but their suitability depends on their catalytic properties, stability and specificity, as these factors can affect cheese yield and sensorial properties [2] (page 2). Chymosin is deemed to have the highest proteolytic specificity for clotting bovine milk; thus, offering the best yields during cheesemaking [42,43] (page 9). As shown in Table 2, significant differences (Tukey’s HSD test *p* < 0.05, identified with upper case letters) in MCIs were observed when using the same coagulant for different milk types. Cr showed the highest MCI values at 25 °C in buffalo milk, whereas the best values were obtained in sheep’s milk at the usual milk clotting temperature of 37 °C. The Cc extract showed the MCI highest values in sheep’s milk at 25, 37, 50 and 70 °C, yet under the same conditions, the MCI values in buffalo and goat milks were not significantly different. Cp showed an efficient coagulation activity at lower temperatures (25 and 37 °C) in caprine and bovine milks; this parameter was higher in caprine milk than all other temperatures tested. Fc appeared to be more efficient at 70 °C in goat’s milk and 37 °C in sheep’s milk, while the highest MCI for Fc was obtained for bovine milk at 25 °C.

Few studies have compared the effects of the different coagulants on different types of animal milks and temperatures. Mazorra-Manzano et al. [42] found that using ginger, melon and kiwi extracts for coagulating cow’s milk was related to their protease specificity and incubation temperature. The effects of the different types of plant rennet on milk coagulation varied depending on the temperatures and milk coagulating enzymes used. However, in our opinion, the results obtained were insufficient to thoroughly evaluate the coagulation efficiency of the plant extracts. The clotting time method applied is somewhat subjective because it relies on ‘‘human eye identification’’ of flocculation, which greatly affects the precision of the observation [44], which is why the ability of different types of milk sources to react with the three plant coagulants and to form a curd with proper consistency in optimal processing time was determined by lactodynamograph [29,45] (page 6). The results obtained are given and discussed below.

### 3.2. Lactodynamographic Parameters (LAT) of Plant Extracts

Milk with favourable coagulation characteristics, such as short coagulation time and firm curd, is usually expected to give higher cheese yield [46]. The methods used for detecting these are based on the use of the lactodynamograph method which measures the physicochemical changes that occur in milk during rennet coagulation [21,47,48] (page 3). The rennet-induced coagulation of milk can be divided into two stages. The first stage is enzymatic hydrolysis during which the renneting enzyme separates caseinomacropeptides from κ-casein, resulting in the disappearance of resistance to the aggregation of casein micelles. The second stage is aggregation, during which casein micelles are assembled into a three-dimensional network that binds moisture, milk fat and other milk solids. The first stage of rennet coagulation is described by a parameter called rennet coagulation time (r, min), which is the time that elapses between the addition of rennet to the milk and the beginning of coagulation. In order to estimate the efficiency of the second stage of rennet coagulation, different coagulation parameters were used: (1) the time (min) that elapsed from adding rennet to the milk or the start of curd formation until a curd firmness of 20 mm (k20, min) was achieved; (2) curd firmness after a certain length of time, which in our case was 60 min (a60, mm) from the start of curd formation.

The LAT parameters measured at 35 °C for cardoon, *F. carica* latex and animal coagulants, in bovine, buffalo, goat and sheep milks, are shown in Table 3. The data regarding the Cp extract are missing since milk coagulation did not occur in any milk sample. Consequently, the resulting clotting properties did not follow a consistent pattern to detect r, k20 and a60. Nevertheless, the two-way ANOVA analysis (milk animal sources versus coagulants) revealed that milk and coagulant factor (*p* < 0.01, data not shown) significantly affected the mean of the r and a60 parameters. Moreover, the LAT parameters analysed using one-way ANOVA enabled us to test for significant differences (Tukey’s HSD test *p* < 0.05, identified with the small letters) between the types of milk according to each coagulant. As regards clotting times (r), no significant differences were observed for Cr added to all types of milk. Interestingly, Cc showed the lowest r values in buffalo milk, followed by goat and sheep milks, while a longer clotting time was required for bovine milk. Fc also showed differences at the onset of milk coagulation, which occurred faster in sheep milk than in buffalo milk. However, *F. carica* latex did not reach curd firmness in buffalo and goat milk, resulting in the unavailability of LAT parameter measurements. As regards the k20 value, no significant differences were observed for Cc, Cr and Fc in the milk samples, except for when Fc was added to sheep’s milk, which then obtained the lowest value for curd firmness. The a60 parameter measured for Cr was not significantly different in buffalo, goat and sheep milks. The same result was obtained for Cc in all milk samples. A higher a60 value was obtained for Fc in sheep’s milk than bovine milk. Moreover, a significant interaction between coagulant according to each milk sample was also observed by one-way ANOVA (Tukey’s HSD test *p* < 0.05, identified with the upper letters). The *r*-values for both Cc and Fc coagulants were significantly lower than for calf rennet in each type of milk. Curd firmness at 60 min proved to be higher in bovine milk incubated with Cr and Fc coagulants.

Curd firmness is determined by both the composition and structure of casein micelles and also by how the caseins are hydrolysed [49]. Calf rennet and cardoon extracts gave similar curd firmness (a60) values for particular milk substrates (buffalo, goat and sheep) suggesting that the two coagulants hydrolyse caseins in a similar manner, especially in the initial stages of clotting, as described in an earlier study [23,50] (page 3). Overall, the clotting properties obtained for plant extracts and calf rennet in sheep’s milk indicate that proteases from plant sources can serve as an alternative to calf rennet in cheesemaking. The fact that Fc incubated at 35 °C clotted buffalo and goat milk indicates that it is not particularly suitable as a coagulant for cheesemaking under these processing conditions.

## 4. Conclusions

Among the plant extracts compared, *C. cardunculus* extracts showed the highest potential for use as a milk-clotting agent in cheesemaking, since it produced a curd with similar characteristics to those obtained using commercial chymosin. *F. carica* latex only appears to be a promising alternative coagulant for curdling bovine and sheep milks. Papain showed the highest proteolytic activity and poorest milk coagulation ability in the different milk substrates. This result also supports the original hypothesis of this study, that the excessive proteolytic nature of plant coagulants can negatively affect the cheesemaking process. This opens a window for deeper investigation on proteolytic activity by applying different coagulation temperatures. Gaining additional insight into this process may enhance the predictability of the clotting efficiencies exhibited by plant rennets in different types of milk and lead to the optimization of their use in the cheesemaking process.

## Figures and Tables

**Table 1:** Chemical compositions of the milk samples used for the coagulation experiments.

	Protein (%)	pH	Casein (%)	Fat (%)	Lactose (%)
Bovine	3.74 (±0.01)	6.58 (±0.01)	2.89 (±0.01)	4.1 (±0.02)	4.62 (±0.08)
Buffalo	4.15 (±0.01)	6.71 (±0.01)	3.21 (±0.01)	7.5 (±0.05)	4.74 (±0.02)
Goat	3.74 (±0.01)	6.65 (±0.01)	2.74 (±0.01)	4.0 (±0.04)	4.51 (±0.01)
Sheep	6.90 (±0.03)	6.34 (±0.01)	5.54 (±0.03)	7.0 (±0.07)	4.45 (±0.01)

**Table 2:** Milk clotting indexes (MCIs) obtained for *C. cardunculus* exctract (Cc), *C. papaya* (Cp), Calf rennet (Cr) and *F. carica* (Fc) latexes, in bovine, buffalo, goat and sheep milks, at different incubation temperatures (25, 37, 50, 60 and 70 °C).

Coagulants	Temperature (°C)	Milk Samples			
		Bovine	Buffalo	Goat	Sheep
Cc	25	3.47 ^dC^ (±0.25)	16.10 ^bB^ (±2.01)	12.49 ^cB^ (±2.50)	35.04 ^bA^ (±2.00)
	37	5.46 ^γ^^B^ (±0.34)	10.61 ^βB^ (±1.51)	8.33 ^χ^^B^ (±2.08)	25.24 ^bA^ (±3.03)
	50	17.51 ^hD^ (±0.27)	24.11 ^hB^ (±2.01)	18.41 ^hB^ (±3.08)	41.40 ^hA^ (±3.50)
	60	21.17 ^η^^D^ (±0.14)	73.28 ^η^^C^ (±3.04)	110.33 ^η^^A^ (±10.02)	87.72 ^η^^B^ (±3.04)
	70	11.21 ^xC^ (±0.252)	36.21 ^xB^ (±2.03)	37.39 ^xB^ (±5.05)	82.31 ^xA^ (±3.05)
Cp	25	23.38 ^cB^ (±0.12)	N/A	49.32 ^bA^ (±4.04)	N/A
	37	39.83 ^βB^ (±0.24)	N/A	88.96 ^βA^ (±4.57)	N/A
	50	3.65 ^jB^ (±0.15)	N/A	6.40 ^hA^ (±0.26)	3.24 ^kB^(±0.31)
	60	10.10 ^φ^^B^ (±0.10)	N/A	10.12 ^φ^^A^ (±3.01)	4.71 ^κ^^B^ (±0.30)
	70	3.52 ^zB^ (±0.23)	N/A	6.53 ^xA^ (±2.50)	2.23 ^yB^ (±0.30)
Cr	25	314.17 ^aD^ (±1.04)	862.92 ^aA^ (±20.63)	371.92 ^aC^ (±10.54)	716.74 ^aB^ (±30.53)
	37	327.62 ^α^^C^ (±2.51)	596.61 ^α^^AB^ (±25.15)	551.67 ^α^^B^ (±25.17)	625.09 ^α^^A^ (±25.00)
	50	447.75 ^gB^ (±2.54)	515.43 ^gA^ (±15.02)	244.86 ^gC^ (±15.00)	540.93 ^gA^ (±40.03)
	60	2311.78 ^γ^^A^ (±7.60)	878.43 ^γ^^D^ (±25.70)	1428.47 ^γ^^C^ (±42.40)	1839.26 ^γ^^B^ (±40.02)
	70	2532.74 ^wA^ (±16.06)	200.00 ^wC^ (±25.17)	1564.46 ^wA^ (±41.08)	1528.77 ^wA^ (±30.08)
Fc	25	56.42 ^bA^ (±2.13)	N/A	N/A	19.82 ^cB^ (±3.02)
	37	24.26 ^χ^^A^ (±2.05)	N/A	N/A	23.05 ^β^^A^ (±3.00)
	50	19.90 ^hB^ (±1.65)	28.68 ^hA^ (±3.51)	1.14 ^jC^ (±0.21)	21.18 ^jB^ (±2.02)
	60	13.23 ^ηφ^^B^ (±2.04)	27.32 ^φ^^A^ (±3.05)	2.90 ^φ^^C^ (±0.40)	16.46 ^φ^^B^ (±2.50)
	70	18.08 ^xB^ (±2.00)	35.51 ^xA^ (±3.12)	5.63 ^yB^ (±0.35)	14.96 ^yB^ (±3.00)

Results are mean values of triplicate experiments with standard deviations. Values with different lowercase letters differ significantly (Tukey’s test, *p* < 0.05) considering the coagulant factor for the single milk animal source incubated at each temperature (25 °C (a, b, c, d), 37 °C (α, β, γ, δ), 50 °C (g, h, j, k), 60 °C (γ, η, φ, κ), 70 °C (w, x, y, z)). Values with different capital letters (A, B, C, D) differ significantly (Tukey’s test, *p* < 0.05) according to milk factor at each coagulant. N/A: not available (coagulation has not been revealed).

**Table 3:** Lactodynamographic (LAT) parameters measured for *C. cardunculus* (Cc), calf rennet (Cr) and *F. carica* (Fc) extracts in bovine, buffalo, goat and sheep milks at 35 °C.

LAT Parameters	Coagulant	Milk			
		Bovine	Buffalo	Goat	Sheep
r	Cr	12.22 ^aA^ (±1.28)	15.22 ^aA^ (±0.53)	10.08 ^aA^ (±0.97)	11.58 ^aA^ (±0.84)
	Cc	42.30 ^aC^ (±0.54)	18.11 ^bA^ (±0.13)	39.27 ^aB^ (±0.87)	21.46 ^bB^ (±1.46)
	Fc	22.9 ^aB^ (±3.15)	N/A	N/A	10.05^bB^ (±0.68)
k20	Cr	9.72 ^aA^ (±2.58)	4.95 ^aA^ (±0.81)	14.37 ^aA^ (±1.71)	4.87 ^aA^ (±0.52)
	Cc	10.01 ^aA^ (±0.71)	6.24 ^aA^ (±0.75)	8.38 ^aA^ (±0.84)	6.34 ^aA^ (±0.80)
	Fc	13.39 ^aA^ (±0.19)	N/A	N/A	8.35 ^bA^ (±1.01)
a60	Cr	54.18 ^aB^ (±1.22)	52.06 ^aA^ (±4.22)	45.20 ^aA^ (±8.63)	58.84 ^aA^ (±3.8)
	Cc	29.45 ^cA^ (±2.22)	59.55 ^aA^ (±1.47)	38.89 ^bA^ (±2.60)	52.77 ^aA^ (±0.87)
	Fc	37.30 ^bB^ (±2.77)	N/A	N/A	53.41 ^aA^ (±3.81)

Abbreviations are: r (min)—clotting time; k20 (min)—curd firming time; a60 (mm)—curd firmness at 60 min after commencement of clotting. The results are mean values of triplicate experiments with standard deviations. Values with different upper letters differ significantly (Tukey’s test, *p* < 0.05) considering the coagulant factor for each type of milk (a, b, c). Values with different capital letters (A, B, C) differ significantly (Tukey’s test, *p* < 0.05) according to milk factor of each coagulant. N/A: not available (firmness was not reached).
